# Effect of feeding garlic leaves on rumen fermentation, methane emission, plasma glucose kinetics, and nitrogen utilization in sheep

**DOI:** 10.1186/s40781-017-0139-3

**Published:** 2017-06-26

**Authors:** Arvinda Panthee, Ayana Matsuno, Mohammad Al-Mamun, Hiroaki Sano

**Affiliations:** 1 Department of Animal Science, Faculty of Agriculture, Iwate University, Ueda 3-18-8, Morioka, Iwate prefecture 020-8550 Japan; 2 Department of Animal Nutrition, Bangladesh Agricultural University, Mymensingh, Bangladesh

**Keywords:** Garlic leaves, Rumen, Glucose kinetics, Methane emission, Nitrogen utilization, Sheep

## Abstract

**Background:**

Garlic and its constituents are reported to have been effective in reducing methane emission and also influence glucose metabolism in body; however, studies in ruminants using garlic leaves are scarce. Garlic leaves contain similar compounds as garlic bulbs, but are discarded in field after garlic bulb harvest. We speculate that feeding garlic leaves might show similar effect as garlic constituents in sheep and could be potential animal feed supplement. Thus, we examined the effect of freeze dried garlic leaves (FDGL) on rumen fermentation, methane emission, plasma glucose kinetics and nitrogen utilization in sheep.

**Methods:**

Six sheep were fed Control diet (mixed hay and concentrate (60:40)) or FDGL diet (Control diet supplemented with FDGL at 2.5 g/kg BW^0.75^ of sheep) using a crossover design. Methane gas emission was measured using open-circuit respiratory chamber. Plasma glucose turnover rate was measured using isotope dilution technique of [U-^13^C]glucose. Rumen fluid, feces and urine were collected to measure rumen fermentation characteristics and nitrogen utilization.

**Result:**

No significant difference in rumen fermentation parameters was noticed except for rumen ammonia tended to be higher (0.05 < *P* < 0.1) in FDGL diet. Methane emission per kg dry matter ingested and methane emission per kg dry matter digested were lower (*P* < 0.05) in FDGL diet. Plasma glucose concentration was similar between diets and plasma glucose turnover rate tended to be higher in FDGL diet (0.05 < *P* < 0.1). Nitrogen retention was higher (*P* < 0.05) and microbial nitrogen supply tended to be higher (0.05 < *P* < 0.1) in FDGL diet.

**Conclusion:**

FDGL diet did not impair rumen fermentation, improved nitrogen retention; while absence of significant results in reduction of methane emission, glucose turnover rate and microbial nitrogen supply, further studies at higher dose would be necessary to conclude the merit of FDGL as supplement in ruminant feedstuff.

## Background

Antibiotics have been used in ruminant and non-ruminant diets since 1940s at sub-therapeutical level for better growth performance [1, 2]. However, the use of antibiotics as growth promoter in animal feed is limited nowadays due to increasing public concerns over the spread of antibiotics resistance in bacterial pathogens, which poses threat to public health. Thus, there has been a considerable research on finding natural alternatives to antibiotics with growth promoting effects [1–4]. In recent years, many researchers are attracted toward plant bioactive compounds for its potential role as substitute to growth promoting antibiotics [1, 3, 5].

Garlic contains numerous active metabolites such as sulfur compounds (thiosulfinates, allyl sulfides, glutamyl cysteines, allicin), enzymes, free amino acids, sterols, steroids, triterpenoid, glycosides, flavonoids, phenols, organoselenium compounds and are also rich in vitamins (especially vitamins of B complex and vitamin C) [6, 7]. Martin et al. [6] reviewed the role of major sulfur compounds in garlic for numerous medicinal properties, such as anticancer, cardioprotective and immunomodulatory, antimicrobial, antioxidant, cardioprotective and immunomodulatory activities. In this regard, in vivo studies in sheep on plasma glucose concentration using garlic oil [8, 9] or raw garlic [9, 10]; on methane emission using allicin [11], garlic oil [12], raw garlic [13] or garlic powder [14]; on performance and carcass characteristics of using garlic bulb and husk [15] has been carried out. Similarly, in-vitro studies by Busquet et al., [16], Soliva et al., [17] showed that garlic oil resulted in stronger effects in methane reduction when compared with effects of each active compound individually and suggested that effects observed are due to synergistic action of compounds present. Thus, instead of using individual components or combined form, using the components as available in plant as such would be beneficial.

Alliin (cysteine sulfoxide) is the major naturally occurring compound present in garlic and gets converted to allicin in presence of enzyme alliinase when crushed [7]. Allicin is unstable and gets converted to various diallyl sulfides [7]. The leaf is primary site for synthesis of alliin from where it gets transferred to other plant parts such as bulb [18]. Alliin concentration was 3.7 mg/g in garlic bulb and 2.79 mg/g in garlic leaves at fresh weight basis in our preliminary experiment. Garlic leaves contains same major bioactive components as garlic bulb [19]. The chemical composition of sulfur–containing compounds found in essential oil isolated from green garlic leaves showed that diallyl trisulfide (32%), methyl diallyl disulfide (31%) and methyl allyl trisulfide (11%) as major components [20] which is similar to garlic bulb essential oil composition [21]. Similarly, the yields of essential oil (*w*/w) was highest on freeze dried compared to air and oven dried process [21]. The loss in the yield was supposed to be due to long drying time required for process, resulting in greater degree of evaporation of the volatile compounds [21] making freeze drying process as most suitable method for preservation of active constituents present in garlic leaves.

Takko city alone in Aomori prefecture in Japan produces about 70% of total garlic production in Japan [22]. Green garlic leaves and stem after harvest of garlic bulb in these areas is left over in field, which causes garlicky stench in the atmosphere. In this scenario, garlic leaves could be of importance as a by-product due to its abundance and possible utilization as livestock feed. Ensiling garlic leaves could be cost effective, as carried out by Kamruzzaman et al. [2], however they concluded that ensiling might have deteriorated the secondary metabolites present in garlic leaves and thus losing its effectiveness. Freeze drying is considered inefficient in terms of energy required for drying, but it has its advantages in terms of preserving the plant bioactive compounds. Keeping aside the cost of freeze drying, current study focuses only on effect of feeding FDGL on rumen fermentation parameters, methane emission, nitrogen metabolism and glucose kinetics in sheep.

## Methods

### Plant material

The garlic leaves (*Allium sativum* var. Fukuchi white) were obtained from the commercial garlic farm in Aomori prefecture, Japan. Dead, dry wilted shoots and leaves were removed and only the green healthy leafy portion was used for the experiment. Garlic leaves were cut in about 2–3 cm in length and freeze dried (EYELA freeze dryer FD/5 N, Tokyo Rikakikai Co., Ltd., Tokyo, Japan). The freeze dried leaves were further grinded and passed through 1 mm screen (Cyclotec ™-1903, Foss Tecator, Sweden) and fed to animal by mixing with concentrate fraction of feed.

### Animals, diet and management

Six healthy crossbred (Corridale × Suffolk) wether of 2 years of age weighing 46 ± 1.2 kg of body weight were used. The experimental design, animal handling, and sample collection were approved by the Animal Care Committee of Iwate University (Approval no. A201532). Two dietary treatments were tested using crossover design with two 22 days periods. Control diet was mixed hay plus concentrate at 60:40 ratio. Mixed hay consisted of orchardgrass (*Dactylis glomerata*) and reed canarygrass (*Phalaris arundinacea*). The chemical compositions of diets are presented in Table 1. The animal received 120 kcal/(kg BW^0.75^·d) metabolic energy, 7.48 g/(kg BW^0.75^·d) crude protein (CP), and 58.68 g/(kg BW^0.75^·d) DM content in control diet. Supplementation of FDGL at 2.5 g/(kg BW^0.75^·d) to the Control diet increased FGDL diet CP content to 7.82 g/(kg BW^0.75^·d) and DM content to 61.18 g/(kg BW^0.75^·d). Body weight measurements were taken weekly and feed was adjusted accordingly. The adaptation period to diet was 14 days in individual sheep pens and during the later 8 days sheep were moved to environmentally controlled house. The control house was maintained at 23 ± 1 °C with lighting from 8:00 to 22:00 h with relative humidity of 70%. Feed was offered once daily at 9:00 h and fresh drinking water was available ad libitum.

**Table 1:** Chemical composition of diets

Chemical composition	Mixed hay^a^	Concentrate	Garlic leaves
Dry matter (DM) (g/kg)	864	888	404
Crude Protein (g/kg DM)	145	152	128
Neutral detergent fiber (g/kg DM)	663	368	598
Organic matter (g/kg DM)	886	919	904
Gross energy (Kcal/g DM)	3.9	3.97	3.55

^a^Mixed hay: mixed hay of orchardgrass and reed canarygrass (60:40)

### Collection of rumen fluid

Rumen fluid (50 mL) was collected on day 21 from each sheep using stomach tube inserted orally before feeding (0 h), 3 and 6 h after feeding. The pH of the ruminal fluid was measured (F-51, Horiba Ltd., Kyoto, Japan) immediately after collection. Rumen fluid was then centrifuged at 5000×g for 10 min at 4 °C (RS-18 IV, Tomy, Tokyo, Japan). Five mL aliquot of the liquid fraction was used to measure ruminal volatile fatty acid (VFA) concentrations and another 1 mL was mixed with 1 mL of 0.1 M HCl for ruminal ammonia (NH_3_) analysis. HCl was added to stop the microbial activity and prevent loss of NH_3_ from ruminal fluid. All samples were kept at −30 °C until further analysis.

### Methane measurement

Sheep were accustomed to open circuit respiratory chambers (50·70·100 cm) prior to the experiment. The methane gas sample from rumen of each sheep released from oral cavity was analyzed using methane gas analyzer (VA 3000A, Horiba Electronics, Kyoto, Japan) from day 15 to day 20, 2 days continuous for each animal. Span gas of 0 ppm and 159 ppm methane was used to calibrate the methane analyzer before the experiment. Fifty L/min of air inside chamber was removed continuously using the pump and a portion was fed to methane gas analyzer. Background air methane gas concentration was measured and subtracted for each sheep. Feed was offered once per day and water was available ad libitum. The temperature, pressure and humidity were recorded simultaneously and converted into standard temperature and pressure conditions and thus final methane volume was standardized.

### Isotope dilution method

An isotope dilution technique using [U-^13^C]glucose was used to measure the turnover rate of plasma glucose on day 22. Two catheters, one for isotope infusion and the other for blood sampling were inserted into left and right jugular veins on the morning of each isotope dilution technique. Catheters were filled with sterile solution of 3.8% tri-sodium citrate in order to prevent blood clotting. Three hours after feeding, 3.0 μmol/kg^0.75^ of [U-^13^C]glucose (D-glucose-^13^C_6_,99 atom% excess ^13^C; Cambridge Isotope Laboratories, USA) dissolved in saline solution (0.9%) was injected as priming dose injection followed by [U-^13^C]glucose infusion at constant rate of 3.0 μmol/(kg BW^0.75^·h) for 4 h using multichannel peristaltic pump (AC-2120, Atto Co. Ltd. Tokyo, Japan). Blood samples were collected immediately before the priming dose and at 30 min interval during the last 2 h of the primed-continuous infusion of [U-^13^C]glucose. The collected blood samples were transferred to heparinized tubes and stored in crushed ice until centrifugation. Blood samples were centrifuged at 8000×g for 10 min at 4 °C and plasmas were then stored at −30 °C until further analysis.

### Nitrogen utilization

Nitrogen utilization trial was carried out for 5 days from day 15 to 19 using metabolic crates. Feces and urine excreted during 24 h period were separately collected. Feces collected was air dried in an oven at 60 °C for 48 h, ground to 1 mm mesh screen and a subsample was stored until further analysis. Urine was collected in a plastic bucket with 50 mL of 3 M H_2_SO_4_. Total volume was measured and a representative subsample was taken. For purine derivatives and microbial protein synthesis, a subsample was diluted five times. Both total urine subsample and diluted urine subsample were stored at −30 °C until further analysis.

### Chemical analysis

Dry matter of feed was determined following the standard procedure of AOAC [23]. Nitrogen content in feed was determined by Kjeldahl method with the Foss Kjeltech System (Tecator Digestor System and Kjeltech 2300, Foss Tecator, Sweden). The neutral detergent fiber was determined according to Van Soest et al. [24] using the Foss Analytical FiberCap system (FiberCap™ 2021/2023, Foss Analytical, Sweden). Concentrations of rumen total VFA were determined by titrating the steam distillate of rumen fluid with 0.1 M NaOH. The titrated distillate was dried and individual VFA concentrations were determined using gas chromatography (HP 5890, Hewlett Packard, USA). The ruminal NH_3_ concentration was determined using colorimetric method as described by Weatherburn [25] using a spectrophotometer (V-630, JASCO, Japan). Isotopic enrichments of plasma [U-^13^C]glucose were measured as described by Fujita et al. [26]. The isotopic enrichments of plasma [U-^13^C]glucose was determined by selected ion monitoring with gas chromatography mass spectrometry system (QP-2010, Shimadzu, Kyoto, Japan). Plasma glucose concentration was determined enzymatically as described by Huggett and Nixon [27].

### Calculation

The purine derivatives excretion, namely allantoin, xanthine plus hydroxanthine and uric acid from diluted urine samples were determined by the procedure described by Chen and Gomes [28]. The amount of microbial purines absorbed (X mmol/d) corresponding to the PD excreted (Ymmol/d) was based Newton’s iteration process [28].

$$ \mathrm{Microbial}\ \mathrm{N}\mathrm{itrogen}\ \mathrm{Supply}\ \left(\mathrm{g}\ \mathrm{N}/\mathrm{d}\right)=70\cdotp \mathrm{X}\ \left(\mathrm{mmol}/\mathrm{d}\right)/0.83\cdotp 0.116\cdotp 1000 $$

Where, digestibility of microbial purines was 0.83, nitrogen content of the purines was 70 mg N/mmol and ratio of purine nitrogen to total nitrogen in mixed rumen microbial biomass was 0.116.

For isotope dilution methods, the turnover rate of plasma glucose was calculated using the equation described by Tserng and Kalhan [29] as follows:

$$ \mathrm{Turnover}\ \mathrm{rate}=\mathrm{I}\times \left(1/\mathrm{E}-1\right) $$

Where, I was the infusion rate of [U-^13^C]glucose and E was the isotopic enrichments of [U-^13^C]glucose during the steady state.

### Statistical analysis

Results were presented as mean values with standard error of the mean. All data were statistically analyzed using analysis of variance with the MIXED procedure of SAS [30]. The least square means statement was used to test the effects of diet and time, with sheep as the random effect. Results were considered significant at the *P* < 0.05 level, and a tendency was defined as 0.05 < *P* < 0.10. The repeated measures statement and the Tukey’s adjustment were used for time course changes and the significance level was *P* < 0.05.

## Result

### Rumen fermentation characteristics

**Fig. 1:**
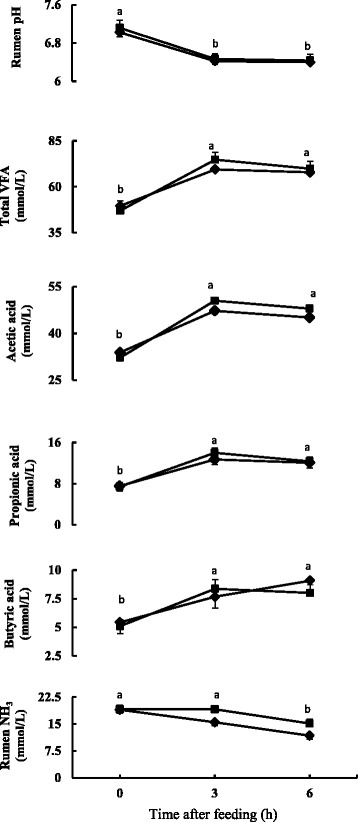
Time course change in rumen pH, total and individual VFA and rumen ammonia in sheep fed Control diet (♦) and FDGL diet (■). Values are expressed as mean ± SEM for *n* = 6. Different letters (*a*, *b*) indicate significant difference between time after feeding

The time course change in rumen fermentation properties is presented in Fig. 1 and the mean values of time course change are presented in Table 2. Rumen pH decreased (*P* < 0.05) after feeding and was similar for three and 6 h after feeding in both the diets. The rumen pH did not differ between the diets and there was no diet and time interaction. The total VFA and individual VFAs concentration increased (*P* < 0.05) after feeding and were similar for 3 and 6 h after feeding. No significant differences were found in total VFA and individual VFAs. The acetic to propionic acid ratio also did not differ between the diets. Rumen ammonia concentration decreased (*P* < 0.05) at 3 h after feeding in Control diet, and in FGDL diet it decreased (*P* < 0.05) at 6 h after feeding (Fig. 1). Rumen ammonia concentration tended to be higher (0.05 < *P* < 0.1) for FDGL diet.

**Table 2:** Dietary effects on rumen pH, concentrations of rumen ammonia and volatile fatty acid in sheep^a^

Item	Diet^b^		SEM^c^	*P*-value		
	Control	FDGL		Diet	Time	**Diet ​× Time**
Rumen pH	6.61	6.67	0.100	0.574	<0.001	0.956
Total VFA (mmol/L)	62.29	63.86	3.543	0.676	<0.001	0.674
Acetic acid (mmol/L)	42.08	43.57	2.195	0.527	<0.001	0.639
Propionic acid (mmol/L)	10.76	11.25	0.772	0.551	<0.001	0.680
iso-Butyric acid (mmol/L)	0.69	0.66	0.091	0.799	0.021	0.485
Butyric acid (mmol/L)	7.39	7.16	0.610	0.713	<0.001	0.349
iso-Valeric acid (mmol/L)	0.87	0.73	0.081	0.231	<0.001	0.289
Valeric acid (mmol/L)	0.49	0.48	0.048	0.857	<0.001	0.548
Acetic/propionic acid ratio	4.02	3.97	0.134	0.706	<0.001	0.335
Rumen ammonia (mmol/L)	15.39	17.78	0.966	0.056	<0.001	0.061

^a^Values represent means of six sheep of before (0 h), 3 and 6 h after feeding

^b^Control diet: mixed hay (orchardgrass and reed canarygrass) and concentrate (60:40); FDGL diet: Control diet plus FDGL

^c^SEM, standard error of mean

### Methane emission (Table 3)

The methane emission per day and methane emission per kg metabolic body weight per day did not differ between diets. The methane emission per kg dry matter ingested (DMI) and methane emission per kg dry matter digested (DMD) was lower (*P* < 0.05) in FDGL diet. Dry matter digestibility did not differ between the diets.

**Table 3:** Effect of feeding FDGL on methane emission in sheep^a^

Items	Diet^b^		SEM^c^	*P*-value
	Control	FDGL		
Methane (L/ d)	25.79	24.38	1.124	0.279
Methane (L/(kg BW^0.75^·d))	1.49	1.40	0.055	0.205
Methane (L/kg DMI)	28.05	25.34	0.919	0.042
Methane (L/kg DMD)	40.79	35.96	1.526	0.034
Dry matter digestibility (%)	68.75	70.56	0.997	0.145

^a^Values represent means of six sheep

^b^Control diet: mixed hay (orchardgrass and reed canarygrass) and concentrate (60:40); FDGL diet: Control diet plus FDGL

^c^SEM, standard error of mean

### Plasma glucose kinetics (Table 4)

**Fig. 2:**
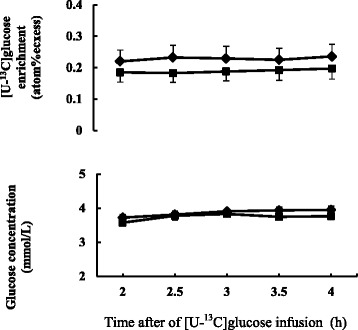
Plasma [U-^13^C]glucose enrichment and plasma glucose concentration in sheep fed Control diet (♦) and FDGL diet (■) during the last 2 h of primed continuous infusion of [U-^13^C]glucose. Values are expressed as mean ± SEM for *n* = 6

No effect was observed on body weight gain between two diets. Basal plasma glucose concentration did not differ between the diets. Plasma glucose concentration and isotopic enrichment of [U-^13^C]glucose were stable during the last 2 h of primed continuous infusion of [U-^13^C]glucose in isotope dilution method (Fig. 2). Plasma glucose turnover rate tended to be higher (0.05 < *P* < 0.1) in FDGL diet compared to the Control diet.

**Table 4:** Effect of feeding FDGL on plasma glucose kinetics in sheep^a^

Items	Diet^b^		SEM^c^	*P*-value
	Control	FDGL		
Body weight gain (kg/d)	0.06	0.09	0.039	0.506
Basal plasma glucose concentration (mmol/L)	3.79	3.68	0.076	0.273
Plamsa glucose turnover rate (mmol/(kg BW^0.75^·h))	1.39	1.59	0.083	0.092

^a^Values represent means of six sheep

^b^Control diet: mixed hay (orchardgrass and reed canarygrass) and concentrate (60:40); FDGL diet: Control diet plus FDGL

^c^SEM, standard error of mean

### Nitrogen utilization

The effect of feeding FDGL on nitrogen utilization is presented in Table 5, and on purine derivative excretion and microbial nitrogen supply in Table 6. The nitrogen intake was higher (*P* < 0.05) in FDGL diet. Fecal nitrogen was lower (*P* < 0.05) in FDGL diet and thus nitrogen absorption was higher (*P* < 0.05) in FDGL diet. Urinary nitrogen did not differ between diets and nitrogen retention was higher (*P* < 0.05) in FDGL diet. The nitrogen digestibility was higher (*P* < 0.05) in FDGL diet. Allantoin excretion tended to be higher (0.05 < *P* < 0.1) in FDGL diet. Uric acid excretion and xanthine plus hypoxanthine did not differ between diets. As allantoin contributes major portion of total purine derivatives excretion, the total purine derivatives excretion in FDGL diet tended to be higher (0.05 < *P* < 0.1). Based on total purine derivative excretion calculations, the total microbial nitrogen supply in FDGL diet also tended to be higher (0.05 < *P* < 0.1).

**Table 5:** Effect of feeding FDGL on nitrogen utilization in sheep^a^

Items	Diet^b^		SEM^c^	*P*-value
	Control	FDGL		
Nitrogen intake (g/(kg BW^0.75^·d))	1.19	1.25	0.001	<0.0001
Fecal nitrogen (g/(kg BW^0.75^·d))	0.34	0.32	0.006	0.027
Nitrogen absorption (g/(kg BW^0.75^·d))	0.85	0.92	0.006	0.001
Urinary nitrogen (g/(kg BW^0.75^·d))	0.44	0.44	0.021	0.068
Nitrogen retention (g/(kg BW^0.75^·d))	0.40	0.48	0.027	0.009
Nitrogen digestibility (%)	71.26	74.06	0.485	0.004

^a^Values represent means of six sheep

^b^Control diet: mixed hay (orchardgrass and reed canarygrass) and concentrate (60:40); FDGL diet: Control diet plus FDGL

^c^SEM, standard error of mean

**Table 6:** Effect of feeding FDGL on purine derivatives excretion and microbial nitrogen supply (MNS) in sheep^a^

Items	Diet^b^		SEM^c^	*P*-value
	Control	FDGL		
Allantoin (mmol/(kg BW^0.75^·d))	0.37	0.43	0.029	0.084
Uric acid (mmol/(kg BW^0.75^·d))	0.03	0.03	0.003	0.441
Xanthine plus hypoxanthine (mmol/(kg BW^0.75^·d))	0.04	0.05	0.004	0.443
Total purine derivatives (mmol/(kg BW^0.75^·d))	0.44	0.52	0.031	0.077
Total MNS (g N/(kg BW^0.75^·d))	0.37	0.43	0.027	0.073

^a^Values represent means of six sheep

^b^Control diet: mixed hay (orchardgrass and reed canarygrass) and concentrate (60:40); FDGL diet: Control diet plus FDGL

^c^SEM, standard error of mean

## Discussion

### Rumen fermentation parameters

The average pH was above six for both diets, thus the rumen environment was favorable for normal fermentation process [31]. The results are consistent with other studies where rumen pH values did not differ on addition of garlic oils [8], garlic components [11] or garlic leaf silage [2]. Conditions where rumen buffering cannot keep pace with accumulation of VFA in rumen, the rumen pH lowers [32]. Low rumen pH for prolonged periods below 5.5 can negatively affect feed intake, microbial metabolism, and nutrient degradation [32]. No such imbalance was found due to addition of FDGL in diet. The observed lower concentration of rumen pH after feeding can be linked to higher VFA concentration in the rumen, which is due to the negative relationship between VFA concentration and pH in rumen fluid [31].

Patra et al. [33] fed garlic bulb at the rate of 1% dry matter intake to buffalo, Chaves et al. [34] fed garlic oil at 0.2 g/kg dry matter to lambs and Wanapat et al. [35] fed graded levels of garlic powder (80–120 g/d) along with treated urea rice straw to steers and did not find any changes in rumen total VFA concentrations. Similar lack of effect was also found by Busquet et al. [16], Klevenhusen et al. [12] and Garcia-Gonzalez et al. [36] on invitro experiments. These findings suggest that inclusion of FDGL, garlic or its components in diet do not have negative effect on carbohydrate fermentation.

Ruminal ammonia nitrogen has been reported to be an important nutrient as this is considered to be the major source of nitrogen for microbial protein synthesis [37]. Wanapat and Pimpa [38] observed higher level of rumen ammonia nitrogen was associated with higher nutrient digestibility and intake. Zhu et al. [39] found an increase in ruminal ammonia when infused garlic oil in rumen at 0.8 g/d in goats. Yang et al. [40] reported that garlic diet increased ruminal ammonia concentration in growing lambs and lactating cows, when garlic fed at 5 g/d. The present experimental findings did not support the findings reported previously who supplemented garlic extract at rate of 15 mg/kg DM invitro [41]; 2 g allicin/d [11], 5 g garlic oil and 2 g DADS/kg DM [12] in sheep and reported decreased ammonia concentration. Wanapat et al. [35] showed that addition of garlic powder did not influence the rumen ammonia concentration in steers. McIntosh et al. [42] observed ammonia concentration to be influenced by dose rate of garlic oil, as in lower concentrations the ammonia concentration did not change or increased but at higher dose, the ammonia concentration decreased due to inhibition of hyper ammonia producing bacteria. Chaves et al., [43] observed that ammonia concentration did not change at 250 mg/L, but increased at 100 mg/L on invitro experiment. The concentration of active compound in garlic leaves might not be high enough to decrease ammonia concentration in our experiment. Furthermore, the excess protein content present in garlic leaves of FDGL diet might also have partly contributed to increase ammonia level.

### Methane emission

Tao et al. [11] supplemented 2 g allicin/day and observed decrease in daily methane output scaled to digestible organic matter intake. Similarly, Patra et al. [13] fed raw garlic at 1% dry matter intake and found reduction of 11% methane emission in sheep fed concentrate-roughage diet at 1:1 ratio in open circuit respiratory chamber. Klevenhusen et al. [12] observed similar reduction in methane emission per kg organic matter digested when fed at 2 g DADS/kg DM in sheep. Klevenhusen et al. [12] speculated that the presence of organic sulfur contributed the growth of anaerobic fungi leading to increased fiber digestibility leading to decrease in methane per kg organic matter digested. In our study, FDGL diet reduced methane per kg dry matter intake was well as per kg dry matter digested without reduction in dry matter digestibility. The antimicrobial activity of garlic has been attributed to the presence of organosulfur compounds, and particularly to the allicin [44], which results change in microbial population in rumen causing the reduction in methane emission [11]. The sulfhydryl groups of the organosulfur compounds found in garlic modify the microbial metabolism through their interaction with the other sulfhydryl groups of microbial protein [45, 46]. The capacity of sulfhydryl containing enzymes participating in different activities of *archea* (methanogens) metabolism [47] decreases methane emission. Miller and Wolin [48] demonstrated by inhibiting 3-hydroxy-3-methyl-glutaryl-CoA reductase, it has the potential to specifically inhibit rumen methanogenic archea without affecting rumen bacteria due to their different membrane lipid composition. Zhu et al. [39] found that the final step of biohydrogenation was interrupted in the rumen of goats infused with garlic oil. We however cannot say that garlic leaves might have similar mode of action because garlic leaves metabolism inside rumen with respect to their anti-methanogenic effects have not yet been identified in detail. Although direct effects against methanogens are probable, indirect effects are also possible through suppression of ruminal protozoa or ruminal fiber degradation or both or lower supply of hydrogen to the methanogens [49].

### Glucose metabolism

Plasma glucose concentration was similar between the diets. This report was in accordance with Chaves et al. [34] and Anassori et al. [9] where plasma glucose concentration did not differ when garlic was fed to growing stage or mature sheep, respectively. Although garlic has long been claimed to possess a hypoglycemic effect [50], attributed due to an increase in serum insulin level [9], we failed to observe the hypoglycemic effect of garlic leaves. Our result contrasts with Kamruzzaman et al. [2], Kholif et al. [51] and Pirmohammadi et al. [10] where feeding garlic silage or garlic components increased basal plasma glucose levels. They suggested the increase might be associated with higher propionate concentration in rumen and propionate is the main precursor of glucose in ruminants. The exact mechanism for increase or decrease in propionate concentration due to garlic constituents have not been discussed elsewhere. In our study, we did not find difference in propionate concentration among the diets which may thus have not been reflected in the plasma glucose level.

To our knowledge no information is reported on the effects of garlic leaves on glucose turnover rate in sheep. The determination of glucose turnover gives more dynamic information about situation of the substrate invivo, rather than the determination of glucose concentration only in plasma. It helps us to determine the glucose recycling in the body i.e. glucose disposal and hepatic glucose production while in a steady state and is measured by using stable isotope using isotope dilution technique. The glucose turnover rate tended to be higher in FDGL diet in our study which might be because of enhancement of glucose utilization by increasing the pancreatic secretion of insulin as reported by Eidi et al. [52] in rats. Anassori et al. [9] observed increased insulin level without rise in blood glucose level when garlic was fed to sheep. Similarly, increased insulin concentration increased glucose turnover rate [53] in both normal and pancreatic dogs. Sano et al. [54] also found that glucose turnover rate increased with increased concentration of insulin in sheep. S-Allyl cysteine sulfoxide and diallyl trisulfide, two major active sulfur compounds of garlic, were shown to have potent insulin secretagogue activity [55, 56]. Furthermore, it has been suggested that these compounds have the effect of sparing insulin form –SH inactivation by reacting with endogenous thiol- containing molecule such as cysteine, glutathione, and serum albumins [55]. In the present experiment, though insulin concentration was not measured, we speculate that the active sulfur compounds present in FDGL might be involved in higher insulin production and thus influencing the turnover rate of glucose.

### Nitrogen utilization

Studies on plant secondary metabolites on ruminants have focused their potential to improve nitrogen utilization as it serves both to solve problem of productivity and environment. Nitrogen retention is the index of protein status in ruminants and microbial protein reaching the duodenum represents the greatest contribution of protein in ruminants [11]. Microbial protein has relatively good amino acid balance, thus it is necessary that it should be maximized for efficient use of feed protein and energy [28]. In our experiment, the nitrogen intake in the FDGL diet was 0.06 g/(kg BW^0.75^·d) higher because of extra nitrogen added by garlic leaves supplement. Fecal nitrogen was lower in FDGL diet because of higher digestibility of N in FDGL diet. Urinary nitrogen was similar between both diets. Nitrogen absorption and nitrogen retention is higher in FDGL diets. Previous studies on nitrogen balance in sheep have shown that garlic or its constituents have shown positive effects. Kamruzzaman et al. [2] fed 10% of hay replaced by garlic leaves and found increased nitrogen absorption, nitrogen digestibility and microbial nitrogen supply in sheep. Tao et al. [11] also found that supplementing 2 g alliin/head/d improved fecal nitrogen, nitrogen retention and nitrogen digestibility in sheep. Wanapat et al. [35] supplemented garlic powder at 40, 80 and 120 g/d did not affect fecal nitrogen, urinary nitrogen was decreased while nitrogen retention was increased only at 120 g/d in steers. The exact mechanism for positive nitrogen balance is yet not clear. Tao et al. [11] proposed the increased digestibility due to increase in cellulolytic bacterial populations. According to Amagase et al. [57] and Kamruzzaman et al. [37] garlic could act as remedy for intestinal disorder, flatulence, worms and respiratory infections. Such positive effect in digestive tract might be one of the reasons for increased digestibility and increased nitrogen balance.

## Conclusions

Inclusion of FDGL as feed supplement at 2.5 g/(kg BW^0.75^·d) had no negative effects on ruminal fermentation characteristics and had positive N utilization. However, research using higher doses of garlic leaves may further explain its potential on methane reduction as well as on glucose turnover rate.

## Abbreviations

DMD: Dry matter digestibility
DMI: Dry matter intake
FDGL: Freeze dried garlic leaves
VFA: Volatile fatty acids
